# The Janus Face of Isoflurane: A Dose–Age-Response Model of Cognitive Impact

**DOI:** 10.3390/biom16081122

**Published:** 2026-08-01

**Authors:** Feng-Yi Hu, Yao Gao, Zi-Xin Liang, Zi-Han Huang, Jin-Long Chang, Dan Cui

**Affiliations:** 1 Department of Physiology and Pathophysiology, School of Basic Medical Sciences, Dali University, Dali 671000, China; hufengyi@stu.dali.edu.cn (F.-Y.H.); gaoyao@stu.dali.edu.cn (Y.G.); liangzixin@stu.dali.edu.cn (Z.-X.L.); huangzihan@stu.dali.edu.cn (Z.-H.H.); 2 School of Nursing, Dali University, Dali 671000, China

**Keywords:** isoflurane, cognitive enhancement, toxicity, dose–age effect, anesthesia

## Abstract

Isoflurane remains a cornerstone of clinical anaesthesia, valued for its robust pharmacological profile. However, its long-term neurological impact, specifically its potential to trigger cognitive impairment, remains a subject of intense debate. As a highly lipophilic agent that readily penetrates the blood–brain barrier, isoflurane exerts complex modulatory effects on various neurotransmitter systems and signaling cascades. While traditional paradigms have predominantly focused on isoflurane-induced neurotoxicity, characterised by neuronal apoptosis, oxidative stress and neuroinflammation, emerging evidence reveals a more nuanced, “Janus-faced” reality. Intriguingly, exposure to low doses during specific developmental stages has been shown to enhance cognitive function and consolidate memory. This suggests that a unidimensional toxicity model fails to capture the drug’s full biological spectrum. In this review, we propose a “dose–age-effect” model, a novel two-dimensional framework designed to reconcile these divergent findings. By categorising isoflurane’s impact across distinct developmental and mature neurobiological stages, we systematically synthesise high-quality evidence ranging from molecular signaling to behavioural phenotypes. We also examine the underlying mechanistic landscape and evaluate potential intervention strategies. This integrated perspective advances our theoretical understanding of anaesthetic–brain interactions and provides a roadmap for optimising clinical protocols and mitigating the risk of adverse postoperative cognitive outcomes.

## 1. Introduction

Preliminary investigations largely supported the hypothesis that isoflurane anesthesia could induce cognitive and memory impairments at different life stages. Nevertheless, these findings exhibit inconsistency. Some researchers have observed cognitive deficits in rodents subjected to anesthesia. These deficits were evidenced through behavioral assessments such as the Morris water maze, novel object recognition, and the Y-maze [1,2,3,4]. A multitude of researchers have documented improvements in cognitive function subsequent to exposure to anesthesia in rodent models [5,6,7]. Despite using identical behavioral methodologies, these studies yielded results that were completely antithetical. We propose that the main reasons for this discrepancy are the age of the experimental animals, the isoflurane concentration used, and the anesthesia duration.

Preclinical investigations using male *C57BL/6J* mice have shown that isoflurane affects spatial learning and memory processes associated with the hippocampus in a concentration- and duration-dependent manner [8]. Exposure to low concentrations of isoflurane (0.7%) for a duration of up to two hours results in an enhancement of cognitive function. Mice that underwent this treatment showed better performance in the Morris water maze test. This improvement was associated with an increase in the N-methyl-D-aspartate receptor subtype 2B (NR2B) and a higher ratio of phosphorylated extracellular signal-regulated kinase 1/2 (ERK1/2) to total ERK1/2. Conversely, extended exposure to elevated concentrations of isoflurane (1.4% for four hours) causes notable cognitive deficits [5,9,10,11,12,13]. From a mechanistic standpoint, this adverse effect arises due to apoptosis facilitated by caspase activity. Furthermore, there was a reduction in NR2B expression within this group, accompanied by decreased levels of phosphorylated ERK1/2 [9]. In contrast to the observations made in the low-dose cohort, the cumulative findings indicate that isoflurane’s impact on cognitive function is meticulously modulated by both its concentration and the length of exposure. These critical parameters decisively influence whether its effects are advantageous or neurotoxic. Similarly, the activity of the hippocampal cytochrome c oxidase/IV complex increased in response to elevated concentrations and prolonged exposure to isoflurane. This increase could potentially facilitate iron-mediated deficits in learning and memory related to apoptosis. This phenomenon has also been observed in non-human primates [14,15,16,17,18,19,20,21,22].

The aforementioned findings corroborate the idea that isoflurane influences spatial learning and memory in a manner that depends on concentration and duration. Therefore, it can tentatively be concluded that exposure to a low dose for a short period of time enhances the cognitive function of developing mice, which corresponds to the neonatal stage in humans. It is important to emphasize that this study focused exclusively on two variables—the precise dosage and duration of anesthesia—and evaluated alterations in the expression of a specific set of molecules only. Nevertheless, the mode of action of isoflurane is extremely complex, involving the coordinated regulation of numerous molecular signaling pathways, cell-mediated biological interactions, autophagy, and various other pathological processes. Therefore, this review uses two age-based models—the developmental and mature stages—to create a two-dimensional “dose–age-effect” framework. This comprehensive framework meticulously investigates variations in the effects of isoflurane across different age groups and dosage levels, while also elucidating the underlying mechanisms. This review aims to provide a theoretical basis for improving clinical anesthesia protocols and addressing cognitive disorders related to isoflurane exposure.

## 2. The Janus Face of Isoflurane on Learning and Memory in Rodents: A Critical Role During Development

**Figure 1:**
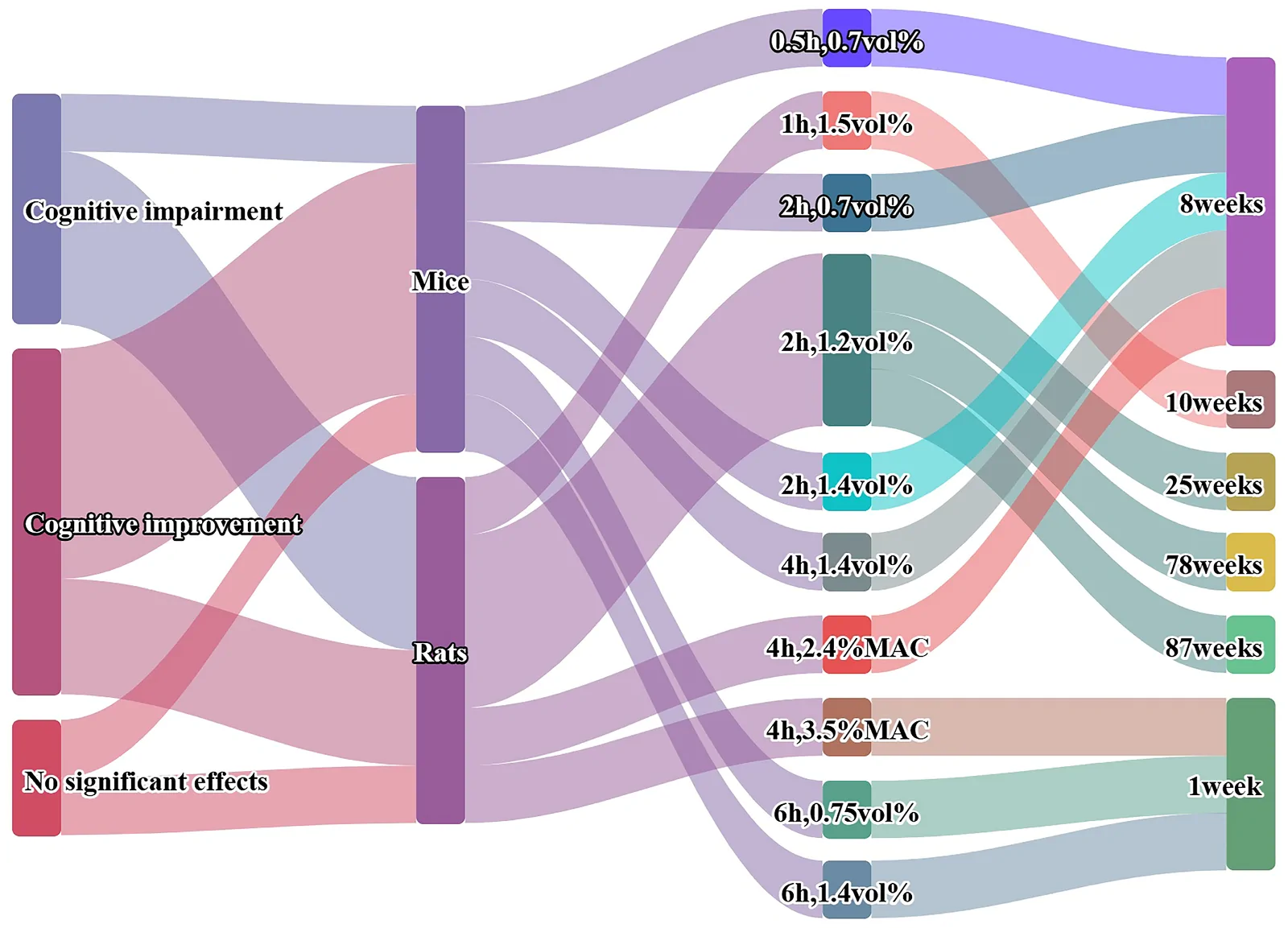
Sankey diagram illustrating cognitive phenotypes following isoflurane exposure in rats and mice. Developmental stage, anesthetic dose, and exposure duration are key factors influencing cognitive function outcomes. For rats and mice of the same age and receiving the same dose, interspecies biological differences act as secondary modifiers, altering the extent and course of cognitive responses (improvement in cognitive function, absence of detectable effects, or cognitive decline). Fluctuating chronic stress merely synergistically exacerbates memory impairment in newborn rats and does not act as a decisive regulator of isoflurane-induced memory impairment.

Isoflurane-induced alterations in learning and memory processes show a Janus face across preclinical studies. Even when the same behavioral tests (Morris water maze, novel object recognition, and conditioned fear) are conducted, researchers often obtain inconsistent behavioral phenotypes. These discrepancies are primarily due to three key experimental factors: the age of the animals, the anesthetic dosage, and the exposure duration. In addition to these primary variables, the existence of species-specific biological differences among rodents is another important confounding factor that exacerbates variability in the results. To systematically distinguish species-specific phenotypes, this study summarized the results obtained in rat and mouse models separately. All behavioral results are categorized in Figure 1.

In rat models, the multifaceted effects of isoflurane on cognitive function are primarily mediated by age and anesthetic exposure parameters, which are the focus of this study. Stratmann et al. [23] demonstrated that long-term exposure to isoflurane has different effects depending on the developmental stage. In young rats at postnatal day 60 (equivalent to human adolescence), four hours of isoflurane administration promoted neuronal differentiation and resulted in long-term neurocognitive benefits. In contrast, in newborn rats at P7, the same exposure regimen suppressed the proliferation of hippocampal progenitor cells and caused delayed, progressive, and persistent hippocampal dysfunction [23]. These age-dependent changes in neural progenitor cells underlie the manifestation of long-term cognitive effects following anesthesia.

Culley et al. also investigated the age- and dose-dependent combined effects of isoflurane in rats [6]. Administration of 1.2% isoflurane by volume for 2 h—a clinically relevant dosing regimen—improved spatial memory in young rats, whereas exposure to the same anesthetic in older rats induced cognitive impairment that persisted for at least 3 weeks. A 2 h administration of 1.2% isoflurane by volume improves cognitive function in 25-week-old rats, but in 78-week-old rats, the same exposure causes severe cognitive impairment [6]. In 10-week-old rats, one hour of exposure to 1.5% isoflurane by volume suppresses long-term potentiation (LTP) and increases GABAergic transmission in the basolateral amygdala (BLA) to baseline levels. This exposure also induces emotional disturbances and impairments in fear learning [24]. Exposure to 1.2% isoflurane for two hours had no significant effect on learning, behavioral activity, or motivation in 87-week-old rats [25].

It is worth noting that none of the aforementioned rat experiments included an intervention involving chronic variable stress (CVS). These consistent observations indicate that age and the dose and duration of anesthesia alone are sufficient to induce bidirectional cognitive phenotypes, regardless of concurrent stress exposure. Although dose and exposure duration partially explain the inconsistencies among the rat study results, developmental stage is a key moderator of isoflurane’s neurocognitive effects. Even when the concentration of the anesthetic and the exposure time are identical, rats of different ages may exhibit completely opposite behavioral outcomes. This supports the notion that age modulates isoflurane neurotoxicity independently. Therefore, a comparative analysis of cognitive function in developing, adult, and aged rat populations optimizes the theoretical framework of the “dose–age-effect” model (see Table 1).

As with the findings observed in rats, the extent of isoflurane-induced memory impairment in mice is primarily determined by developmental stage and anesthetic dose. Conversely, species-specific neural circuit characteristics and fluctuating chronic stress do not significantly contribute to cognitive impairment. They act solely as secondary synergistic modifying factors.

In the absence of stress-inducing interventions, exposure to low doses of isoflurane during the neonatal period results in the persistent restructuring of fear-related memories. Sun et al. reported that exposing one-week-old newborn rats to 0.75% isoflurane by volume for six hours promotes the consolidation of fear memories in adulthood by activating the dorsomedial thalamus and modulating γ-wave oscillations [26]. Even at 8 weeks postnatal, conflicting cognitive outcomes were observed solely due to differences in isoflurane concentration and exposure duration. Exposure to 0.7% isoflurane by volume for 0.5 h positively regulated the expression of NR2B and p-ERK1/2, thereby enhancing cognitive performance. Prolonged exposure (four hours at 1.4% by volume), on the other hand, elevates cystatin levels, inhibits NR2B and p-ERK1/2 signaling, and induces a significant decrease in cognitive function. Under moderate dosing conditions, no significant changes in cognitive function were observed [9]. These results confirm that, in the absence of stress, age and dose independently determine changes in cognitive function in rats.

Fluctuating chronic stress acts as a synergistic aggravating factor only in an anesthetic model using newborn mice. It cannot induce cognitive impairment without concurrent exposure to isoflurane. Zhong et al. [27] demonstrated that the combination of neonatal isoflurane exposure and CVS causes cumulative abnormalities in neuroplasticity. This combination increases the accumulation of ΔFosB in the lateral temporal lobe (BLA) and the dentate gyrus (DG) of the hippocampus. It also alters c-Fos expression and inhibits fear extinction [27]. From a mechanistic perspective, the simultaneous occurrence of anesthesia and stress during the neonatal period results in the reorganization of the neural circuits responsible for fear processing, resulting in a persistent behavioral phenotype resembling anxiety. All stress-related cognitive impairments are observed only in specific subgroups of newborn mice and are not seen in experiments using other mice. This indicates that stress is not an essential regulatory factor in interpreting isoflurane-mediated memory impairments.

In rats, exposure to isoflurane during the developmental period induces persistent changes in fear-related behaviors through multiple pathways. These pathways include remodeling of the amygdala–hippocampus circuit and modulation of gamma-wave oscillations in the thalamus. Detailed experimental parameters and behavioral phenotypes from all studies using rats are summarized in Table 2.

## 3. Dose-Dependent Mechanisms of the Cognitive Effects of Isoflurane: Differences Between Developmental and Adult Stages

The aforementioned studies have illuminated the intricately linked bidirectional cognitive impacts of isoflurane, which are closely associated with both age and dosage levels. This phenomenon may be explained by the distinct ways different dosages of isoflurane influence neuronal activity, signaling pathways, and the neural microenvironment at different stages of development. Moreover, although high and low dosages engage common mechanistic pathways, they also manifest age-specific variances. This section will provide a thorough, systematic analysis of the molecular mechanisms by which isoflurane affects cognitive function. Our analysis will encompass developmental stages and adulthood. It will also explore the differential effects of low and high dosage exposure.

For this review, low-dose isoflurane usually refers to subanesthesia or low anesthetic concentration (about 0.7–1.2%), while high-dose exposure refers to concentrations that reach or exceeds the conventional anesthesia range (about 1.4–2.0%) [5,9]. However, these classifications should not be interpreted as strict pharmacological thresholds, because the cognitive consequences of isoflurane are strongly influenced by exposure time, developmental stage and species. The concentration–effect relationship is not always linear [6,9,23]. Therefore, this review adopts a framework based on the main neurocognitive results reported in the literature.

### 3.1. Dose-Dependent Mechanisms of Isoflurane in the Developing Brain

#### 3.1.1. High-Dose Exposure During Development: Mechanisms of Neurotoxicity and Cognitive Impairment

Research indicates that early exposure to anesthesia may adversely affect an animal’s neurological, social, and emotional development [28]. In the context of oxidative stress, isoflurane impairs the functionality of the mitochondrial electron transport chain, increases the generation of reactive oxygen species (ROS), and compromises the integrity of the glutathione system. Isoflurane accelerates the degradation of the antioxidant enzyme glutathione peroxidase 4 (GPX4) through a heat shock protein HSP90-dependent chaperone-mediated autophagy mechanism. This process removes the suppression of lipid peroxidation [29]. At the same time, isoflurane inhibits the expression of solute carrier family 7 member 11 (SLC7A11), an integral component of the Cystine/Glutamate Antiporter System (Xc^−^ system). This results in the depletion of glutathione, subsequently compromising cellular antioxidant defenses [14]. The aforementioned modifications collectively lead to the accumulation of lipid peroxides, ultimately triggering ferroptosis. Consistently, the application of HSP90 inhibitors, ferroptosis inhibitors, or mitochondrial protectants can markedly mitigate neurological injury and cognitive impairments. Notably, at elevated concentrations of isoflurane, neuronal damage is further characterized by necroptosis and the activation of pyroptosis—a phenomenon substantiated in neonatal piglets [30], newborn non-human primates [31], and the frontal cortex of fetal sheep [32]. Isoflurane triggers necroptosis by relieving the inhibition of Receptor-Interacting Protein Kinase 1 (RIPK1). This releases damage-associated molecular patterns, which markedly activates glial cells and establishes a persistent inflammatory feedback loop [33]. At the same time, isoflurane can activate the NOD-like receptor pyrin domain containing 3 (NLRP3) inflammasome in microglia. This process initiates pyroptosis and releases large amounts of pro-inflammatory cytokines. This amplification of neuroinflammation and synaptic injury intensifies the deleterious effects on neural tissues [33,34]. Elevated levels of isoflurane are more prone to result in enduring challenges with cognitive learning capacity and memory retention.

In addition to directly triggering cell death, high-concentration exposure can extend its deleterious effects through epigenetic mechanisms. For instance, isoflurane enhances the expression of Histone Deacetylase 2 and 3 (HDAC2/3) and suppresses histone acetylation. Concurrently, it diminishes the expression of Brain-Derived Neurotrophic Factor (BDNF) via the long non-coding RNA BDNF-AS and the MicroRNA(miR)-214-3p axis, ultimately hindering the transcription of genes integral to synaptic plasticity [35]. Furthermore, inhibiting the vascular endothelial growth factor receptor 2 (VEGFR2) signaling cascade by activating microglia may hinder the maturation of neural stem cells [36]. Evidently, protracted cognitive dysfunction caused by high concentrations of isoflurane stems from the combined effect of several pathological mechanisms. These encompass immediate neuronal apoptosis as well as sublethal alterations, such as epigenetic reconfiguration and disturbances within the neural microenvironment.

In addition to cell death, neuroinflammation, and epigenetic abnormalities, evidence suggests that anesthetics may cause related cognitive disorders by damaging the cytoskeleton. The dynamic reshaping process is closely related to learning and memory formation because it is the core structure of axon transport and synaptic maintenance in neurons [37]. Tau protein is a microtubule-associated protein that stabilizes microtubules. It is now regarded as an important molecular link between exposure to anesthesia and cognitive dysfunction. Studies have found that various inhaled anesthetics can induce abnormal tau protein phosphorylation by regulating kinase/phosphatase systems, such as GSK-3β, CDK5, and PP2A [38,39]. The detachment of abnormally phosphorylated tau protein from the microtubule skeleton can lead to microtubule depolymerization, axon transport disorders, and a decline in synaptic structural stability [38].

Another study found that in young mice models, anesthetic-induced Tau abnormalities can further promote IL-6 release, mitochondrial dysfunction and decreased synaptic expression, ultimately leading to impaired learning memory ability [8]. At the same time, pathological tau protein can be transported from neurons to small glial cells via extracellular vesicles. This process amplifies neuroinflammatory reactions and worsens cognitive impairment [8,40]. Although research on isoflurane is still relatively limited, existing evidence suggests that tau-mediated microtubule skeletal instability may be an important downstream mechanism connecting neuroinflammation, mitochondrial damage, and synaptic dysfunction. This mechanism may also play a role in the development of long-term cognitive dysfunction following exposure to high-dose anesthesia [38].

It is notable that the cognitive deficits induced by isoflurane exposure during developmental stages can be improved under specific intervention circumstances. Sevoflurane, as an analog of isoflurane with similar pharmacological effects, can serve as a reference for exploring the underlying mechanisms. Studies indicate that consistent physical activity throughout gestation by pregnant rats can remedy the cognitive deficits in their progeny resulting from anesthetic exposure. Exposure to sevoflurane reduces histone acetylation levels in fetal brain and postnatal hippocampal tissue, accompanied by inhibition of the BDNF/TrkB/Akt signaling pathway and a decrease in dendritic spine density [41]. Furthermore, maternal physical activity during pregnancy increases BDNF levels and neuronal counts in the hippocampus of adult offspring, thereby mitigating these adverse effects. Interestingly, this protective influence was absent in the cerebral cortex. Consequently, we propose that cognitive deficits induced by isoflurane and similar anesthetics primarily affect the hippocampus rather than the cerebral cortex during developmental stages [42,43].

#### 3.1.2. Low-Dose Exposure During Development: Cognitive Enhancement and Adaptive Regulatory Pathways

**Figure 2:**
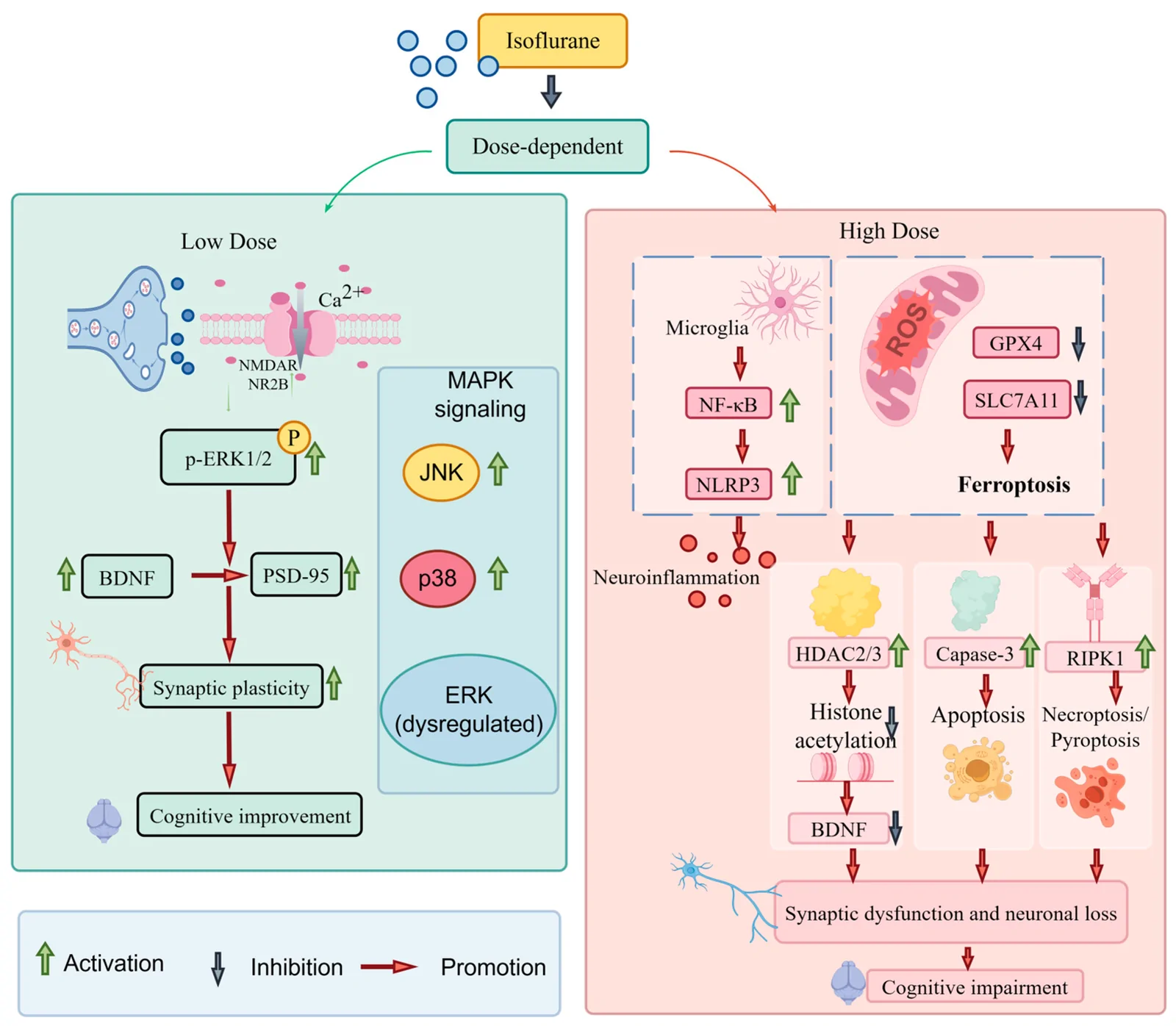
Schematic diagram of the dose-dependent molecular mechanisms underlying isoflurane-induced cognitive effects, presented as a conceptual framework for interpreting the following section. Core molecular mechanisms underlying the dose-dependent cognitive effects of isoflurane. Isoflurane exerts bidirectional effects on cognition through dose-dependent divergence of molecular pathways. At low doses, isoflurane enhances synaptic plasticity by increasing NR2B-containing NMDA receptor activity and activating ERK1/2 signaling, leading to upregulation of BDNF and PSD-95, and ultimately resulting in cognitive improvement. In contrast, high-dose exposure induces neurotoxicity through multiple converging mechanisms. Activation of microglia promotes the NF-κB/NLRP3 inflammatory axis, leading to neuroinflammation. Concurrently, mitochondrial dysfunction and reactive ROS accumulation drive ferroptosis via downregulation of GPX4 and SLC7A11. In addition, caspase-3-mediated apoptosis and RIPK1-associated necroptosis/pyroptosis further contribute to neuronal damage. Epigenetic repression mediated by HDAC2/3 reduces histone acetylation and suppresses BDNF expression. MAPK signaling functions as a context-dependent regulatory node, in which ERK1/2 is primarily associated with adaptive synaptic responses, whereas JNK and p38 are linked to stress and injury pathways. These molecular events converge on synaptic dysfunction and neuronal loss, ultimately leading to cognitive impairment.

Figure 2 illustrates the fundamental molecular mechanism underlying the dose-dependent cognitive effects of isoflurane and serves as a conceptual framework for further discussion.

During the developmental phase, exposure to a low dose of isoflurane continues to activate the c-Jun N-terminal kinase (JNK)/p38 mitogen-activated protein kinase (MAPK) stress signaling pathways while inhibiting the ERK1/2 signaling pathway [44]. At the same time, an inflammatory response mediated by nuclear factor kappa-light-chain-enhancer of activated B cells (NF-κB) may also be partially triggered [45]. Additionally, increased HDAC2/3 expression coupled with reduced histone acetylation levels indicates a shift in epigenetic regulation. The moderate activation of microglia, coupled with the inhibition of VEGFR2 signaling, could potentially impact the development of neural stem cells and the formation of synapses [36]. On an electrophysiological scale, the excitatory effect of δ-subunit-containing GABAα receptors is amplified, refining the excitation–inhibition equilibrium and decelerating gamma oscillations [46]. Concurrently, variations in the expression levels of postsynaptic density protein 95 (PSD-95) suggest compromised stability of the postsynaptic scaffold at excitatory synapses [47].

However, administering a low dosage of isoflurane could potentially enhance cognitive function by facilitating the effective consolidation of fear-related memories. This is in stark contrast to the well-documented detrimental impact that high-dose anesthesia has on neurodevelopment [26]. Studies on developing mice that were administered low doses of isoflurane have demonstrated the potential of isoflurane to enhance fear memory in adulthood. This enhancement is primarily achieved by activating the mediodorsal thalamus and slowing gamma oscillations. When these fear memories are reactivated, the density of c-Fos-positive neurons in the mediodorsal thalamus of mice in the exposure cohort increases significantly [26]. During this time, subtle disturbances in neural activity may alter the connectivity of important nodes, such as the medial dorsal thalamus (MDL). This could lead to better memory performance in adulthood instead of a decline. Furthermore, exposure to isoflurane induces the accumulation of the ΔFosB protein in the basolateral amygdala (BLA) and dentate gyrus (DG) during the early stages of development. This may lead to abnormalities in c-Fos expression and impair the extinction of fear responses [27]. Intriguingly, cerebral ischemic stroke (CIS) has emerged as the second most prevalent cause of mortality on a global scale [48]. Following ischemic-reperfusion injury (CIRI), the administration of isoflurane may enhance autophagy by activating the AMP-activated protein kinase (AMPK)/uncoordinated 51-like autophagy-activating kinase 1 (ULK1) signaling pathway. This activation could subsequently suppress the secretion of inflammatory mediators from the NLRP3 inflammasome [48]. Recent findings present promising therapeutic implications for patients experiencing cognitive impairments associated with CIS, advancing our understanding of neurological function and safeguarding against cognitive decline. However, the underlying mechanisms that distinguish these findings from those related to neurotoxicity are still unclear and require further investigation. Resolving this issue necessitates a detailed examination of the molecular distinctions between afflicted and healthy mice. Although exercise has well-documented benefits for mitigating depression, the impact of isoflurane on spatial learning and memory depends on its concentration and the duration of exposure. A minimal dose of isoflurane (0.5 MAC, 0.7%) and short-term exposure (less than two hours) facilitates hippocampus-dependent spatial learning in young adult mice. This enhancement is achieved by acutely and sustainably upregulating NR2B subunits in hippocampal NMDA receptors and immediately activating the ERK1/2 signaling pathway. Nevertheless, increased isoflurane concentrations (up to 1 MAC, or 1.4%) and prolonged exposure (more than two hours) can lead to the rapid and sustained activation of cleaved caspase-3 and apoptosis of hippocampal neurons. This process inhibits the expression of the NR2B protein and the phosphorylation-driven activation of ERK1/2. This results in the long-term impairment of spatial cognition [9]. Unfortunately, the response threshold is still at an isoflurane concentration of 1.2% and an exposure duration of two hours at this stage of development. To enhance accuracy, it is crucial to conduct further analysis by examining different age groups, dosage levels, and exposure durations.

Additionally, a recent clinical study of various anesthetics reported the potential cognitive benefits of dextromethorphan for children with neurodevelopmental disorders. Since May 2022, the Anesthesiology Department at the Guangzhou Maternal and Child Medical Center has treated 204 pediatric patients with a new type of dextromethorphan nasal spray. Of these patients, 195 were autistic, five had ADNP syndrome, and four had intellectual disability [49]. Nearly 70% of children have improved in core symptoms such as social interaction, language expression and emotional regulation. The observed positive results suggest that low doses of isoflurane (e.g., 1.2% for less than two hours) may produce similar cognitive effects during development under strictly controlled conditions. Due to the fact that its potential neurotoxicity remains a significant concern, this hypothesis is highly speculative and requires thorough verification before it can be considered for clinical use.

In summary, the impact of both low- and high-dose isoflurane on inducing cognitive impairment in developing mice appears to be governed by similar regulatory mechanisms, as illustrated in Table 3.

In order to develop a coherent understanding of these concurrent processes, it is crucial to examine the intersection of adaptive synaptic plasticity and neurotoxic pathways, especially in regard to the dose-dependent molecular divergence induced by isoflurane during developmental stages.

### 3.2. Cognitive Effects and Mechanisms of Isoflurane in the Mature Brain

The influence of isoflurane on cognitive function in mature brains is distinctly related to its concentration and the individual’s age. For the purpose of this analysis, we have delineated the mature stage into two distinct phases: adulthood and early aging. The observed impact patterns between these phases differ markedly, which is indicative of the brain’s evolving capacity to modulate neuroplasticity, as well as the system’s varying degrees of susceptibility.

#### 3.2.1. Cognitive Effects of Low-Dose Isoflurane Exposure During Adulthood

##### Low-Dose Exposure in Adulthood: Reversible Cognitive Impairment

During the Touchscreen Unique Nonmatching-to-Location (TUNL) spatial working memory task with adult rats, administering low-dose isoflurane (approximately 0.3% by volume) resulted in impaired working memory. Notably, this effect was swiftly reversible, indicating the working memory impairment is independent of any loss of consciousness or motor dysfunction [47,50]. In cognitive assessments such as the T-maze, mice exposed to a low concentration of isoflurane (around 1%) demonstrated significant learning impairments [50]. In the Morris water maze assessment, mature mice exposed to 1% isoflurane demonstrated a prolonged path to the exit, signaling a decline in cognitive acquisition abilities. Additionally, this dosage cohort exhibited a heightened propensity for apoptosis within the hippocampal CA1 area, implying that low-concentration isoflurane could potentially result in alterations in learning and memory capabilities, predominantly via compromised hippocampal plasticity [51].

Mechanistically, hippocampal slice studies show that isoflurane at clinically relevant concentrations (0.2–0.3 mM) completely blocks LTP induction in mice CA1 neurons; the blockade is reversible and GABAα receptor-dependent [52]. A similar dose-dependent LTP impairment occurs at concentrations above 2-fold MAC [53]. In cell cultures, isoflurane promotes internalization of NMDA receptors, especially NR2B subunits [54]. In adult mice, it activates calcineurin, lowers ERK phosphorylation, and reduces Egr-1 expression [55]. In addition, isoflurane suppresses the BDNF-TrkB signaling pathway and its downstream ERK/CREB cascade, which further contributes to reduced learning efficiency [56].

##### Low-Dose Exposure in Early Aging: Amplification of Irreversible Cognitive Impairment

The aforementioned effects become increasingly evident during the initial phases of brain aging [6]. Cognitive decline was noted in older mice under identical low-dose exposure conditions, whereas an enhancement in cognitive function was observed in younger mice [57]. Because of its intrinsic structural and molecular vulnerabilities, the aging brain lacks the ability to adapt reversibly when exposed to low concentrations of isoflurane, such as 1%. On the contrary, this initiates a cascading sequence of detrimental processes, each compounding upon the other across various dimensions. These vulnerabilities interact with isoflurane’s neurotoxic properties, resulting in an amplified and prolonged impact that manifests as significantly worsened and persistent cognitive impairment.

#### 3.2.2. Cognitive Effects of High-Dose Isoflurane Exposure During the Maturation Period

##### High-Dose Exposure During Adulthood: Reversible Cognitive Improvement or No Significant Impairment

When mature animals are subjected to elevated concentrations, a variety of distinct effects can be observed. These effects differ from those observed during low-dose exposure in the T-maze task; no significant learning impairments were observed in mice exposed to higher concentrations of isoflurane (e.g., 2%) [50]. In the Morris water maze experiment, adult mice subjected to higher concentration levels (1.5% and 2%) exhibited performance comparable to that of the non-anesthetized control group [51].

Further studies have shown that isoflurane anesthesia can enhance hippocampal LTP by upregulating the expression of the NR2B subunit of the NMDA receptor, and can improve learning and memory performance for a certain period of time. Nevertheless, this phenomenon is evidently reversible and progressively diminishes following the cessation of anesthesia. [5]. Initially, this result seems to contradict the previous discovery that isoflurane can block the induction of LTP in the hippocampus section [52]. However, this difference may be explained by the different experimental conditions. The inhibitory effect is measured when the drug is administered directly to an acute brain section. In contrast, the enhancement effect is observed several hours after recovery from anesthesia, when the NR2B receptor has undergone compensatory upregulation [5]. Therefore, whether isoflurane is inhibited or enhanced LTP may depend on the measurement time, experimental model (in vitro and in vivo) and analysis period. Additionally, exposure to low doses or short durations of isoflurane enhances the spatial learning abilities of adult *C57BL/6J* mice, which correlates with the activation of NR2B and ERK signaling pathways. However, exposure to higher doses or longer durations leads to neuronal apoptosis and cognitive deficits [9].

Notably, the cognitive enhancements resulting from high-dose exposure during adulthood are similar to the cognitive impairments caused by low-dose exposure. Specifically, both effects are temporary and reversible. This suggests that the response of the adult brain to isoflurane is primarily influenced by reversible synaptic plasticity rather than the permanent damage typically associated with aging.

##### High-Dose Exposure in Early Aging: Exacerbation of Irreversible Cognitive Impairment

During the early stages of aging, exposing the brain to high concentrations of isoflurane does not improve cognitive abilities. In fact, it worsens cognitive decline. While the adult brain can preserve its cognitive abilities, occasionally even experiencing temporary improvements, the aging brain suffers pronounced and lasting impairment. This exacerbation is attributed to factors such as diminished protective mechanisms, heightened inflammatory responses, and disturbances to the blood–brain barrier. The aging brain’s structural and molecular vulnerabilities significantly amplify the neurotoxic properties of isoflurane, resulting in more severe and prolonged cognitive impairment.

#### 3.2.3. Integration of Mechanisms Underlying Isoflurane-Induced Cognitive Impairment in the Aging Brain

Investigate the impact of isoflurane exposure on the aging brain, focusing on the subsequent aberrant alterations in intricate molecular pathways.

Isoflurane worsens the disruption of neuroprotective and plasticity-related signaling pathways, especially the ERK/cAMP response element-binding protein (CREB) pathway. This pathway is already impaired in the hippocampus of older individuals. At the same time, it hyperactivates the mammalian target of rapamycin (mTOR) signaling pathway. This results in an amplification of neuroinflammatory responses and a deterioration of synaptic function [58]. Furthermore, existing studies have demonstrated that inhaling anesthetics can exacerbate tau-related pathological changes. This can lead to reduced microtubule stability, impaired intracellular substance transport, and diminished synaptic plasticity [38]. Compared to young brains, aging brains exhibit decreased regulation of protein homeostasis and reduced efficiency in clearing abnormal proteins. This facilitates the sustained accumulation of tau-associated pathology [59]. These cytoskeletal abnormalities can interact synergistically with neuroinflammation, mitochondrial dysfunction, and synaptic damage. This interaction can amplify cognitive impairment following isoflurane exposure. It can also explain the slow or progressive decline in postoperative cognitive function observed in elderly individuals [38]. On an epigenetic scale, isoflurane facilitates the downregulation of miR-365a-3p, consequently relieving its suppressive influence on the MyD88-NF-κB signaling pathway [60], which drives the amplification of inflammatory signaling cascades and leads to neuronal damage. On a metabolic scale, isoflurane exerts an inhibitory influence on cystathionine β-synthase (CBS) activity, consequently diminishing endogenous hydrogen sulfide synthesis. Furthermore, it perturbs protein homeostasis, undermines antioxidant defense mechanisms, and consequently postpones cognitive recuperation [61]. At the systemic level, the blood–brain barrier, which is already compromised in an aging brain, is further damaged by isoflurane. This allows toxic factors from the periphery to enter the brain more easily. Simultaneously, this stimulus enhances β2-microglobulin concentrations, triggers microglial polarization towards the detrimental M1 phenotype, and encourages β-amyloid accumulation, thereby expediting neurodegenerative progression [62].

**Figure 3:**
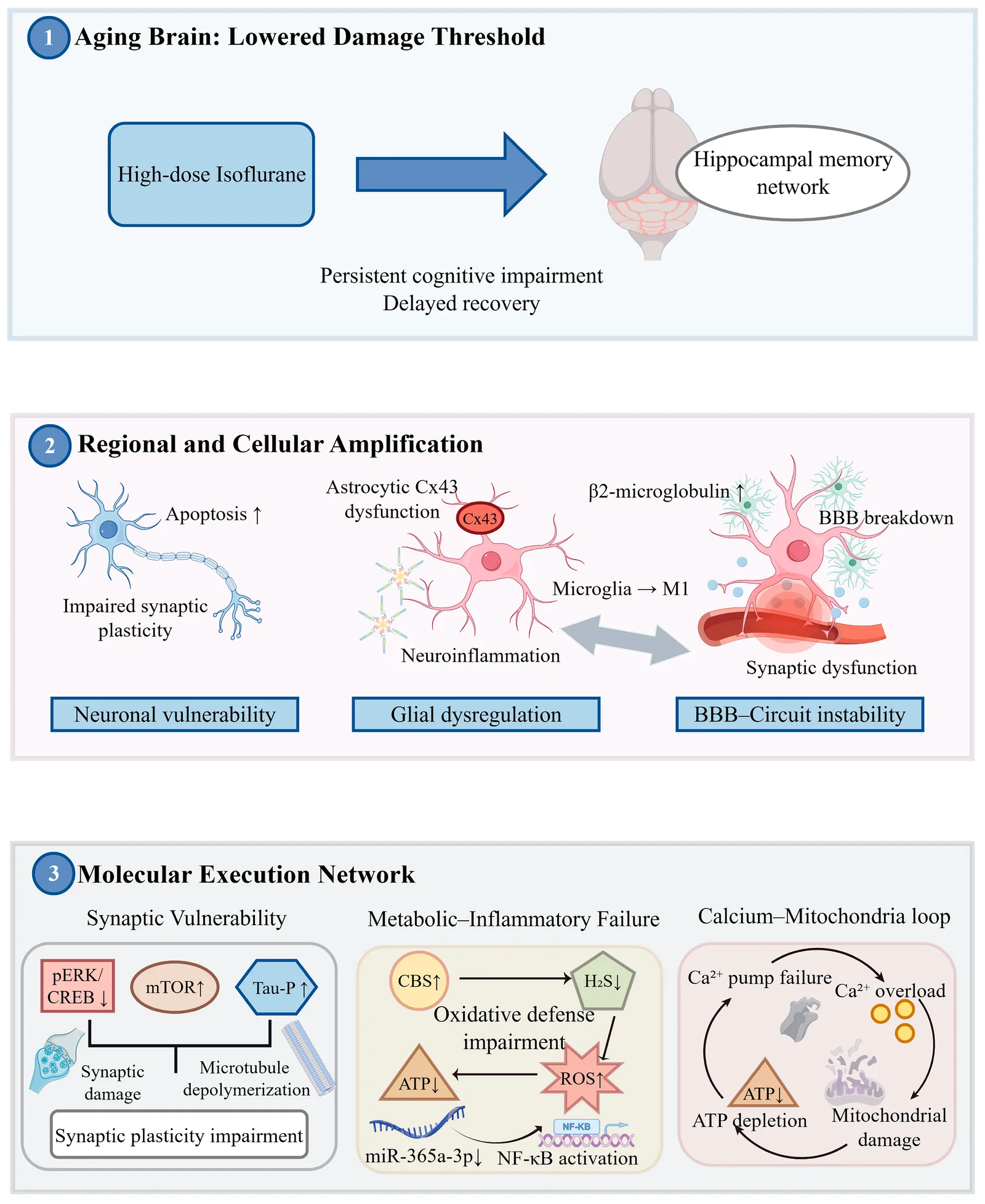
Hierarchical amplification mechanism of cognitive impairment induced by isoflurane in aged brains. Aging lowers the threshold for neural injury, making hippocampal memory networks more vulnerable to high-dose isoflurane exposure, thereby increasing the risk of persistent cognitive impairment and delayed recovery (Panel 1). At the regional and cellular levels, neuronal vulnerability, glial dysregulation, and blood–brain barrier instability amplify local pathological responses, including impaired synaptic plasticity, neuroinflammation, and circuit dysfunction (Panel 2). At the molecular level, multiple interacting pathways contribute to cognitive decline (Panel 3), including synaptic vulnerability characterized by ERK/CREB suppression, aberrant mTOR activation, and tau hyperphosphorylation, which collectively promote synaptic dysfunction and microtubule destabilization; metabolic-inflammatory failure involving CBS/H_2_S deficiency, oxidative stress, NF-κB activation, and ATP depletion; and a calcium–mitochondrial positive feedback loop leading to mitochondrial damage and energy failure. Together, these mechanisms may explain the enhanced sensitivity of the aged brain to isoflurane-induced cognitive deficits. An up arrow indicates an increase or activation, while a down arrow indicates a decrease or inhibition.

Experimental investigations have demonstrated that exposure to isoflurane does not result in cognitive impairment in aged mice when particular experimental conditions are met. These conditions include assessments through maze and rotarod tasks [63]. One plausible explanation for these isolated anomalies is that isoflurane-induced cognitive decline may not affect all aspects of cognitive function equally. Instead, it might preferentially influence tasks predominantly assessing spatial memory processes. The underlying mechanisms responsible for the diverse effects of high-dose isoflurane on the aging brain can be conceptualized using a hierarchical amplification model (refer to Figure 3).

#### 3.2.4. Hierarchical Amplification Model: An Integrative Framework for Multilevel Interactions

The exacerbating impact of isoflurane on age-associated cognitive decline cannot be attributed solely to a single causative factor. Instead, it emerges from the complex interplay of structural and functional modifications across three distinct levels: intracellular, glial, and the blood–brain barrier. These modifications occur alongside interactions within the cellular network. Specifically, the integrity of intracellular defense signaling pathways becomes compromised, leading to a diminished capacity for the brain to shield itself from the deleterious effects of isoflurane exposure. Furthermore, the heightened reactivity of glial cells makes them the main target of isoflurane. This subsequently causes systemic regulatory dysfunction due to compensatory imbalances [64]. The blood–brain barrier’s increased fragility and cellular networks’ decreased stability allow peripheral toxins to encroach and lead to neural circuit dysregulation. Together, these changes transform the aging brain into a “vulnerable system,” making it more susceptible to inflammation and neural network dysregulation, even at standard isoflurane anesthetic dosages. This, in turn, results in irreversible cognitive decline, which is further sustained and aggravated by factors such as the deleterious cycle of mitochondrial damage and energy deficits initiated by calcium overload [65] and compensatory hyperconnectivity in neural circuits [66].

## 4. Multi-Target Interaction Networks

While age-related mechanisms elucidated previously offer explanations for certain behavioral disparities, the multifaceted impact of isoflurane arises from the intricate interplay involving numerous targets. As a volatile anesthetic, isoflurane’s efficacy is mediated through its interaction with various molecular targets. Herein, we will summarize pivotal molecular targets and examine their influence on the progression of neurological disorders, taking into account variations in age and dosage, predominantly in rodent models such as rats and mice.

### 4.1. Age-Dependent Regulation of the Inhibitory System

The modulatory influences of isoflurane on inhibitory neural systems principally involve GABAα receptors and glycine (Gly) receptors—both ligand-gated chloride ion channels. These receptors demonstrate notable concentration-dependent and age-dependent functional and expression characteristics, rendering them crucial targets in understanding the dual cognitive impacts induced by isoflurane.

#### 4.1.1. GABAα Receptors

Isoflurane largely exerts its anesthetic effects by targeting GABAα receptors. It enhances the function of these receptors, demonstrating a particular affinity for those containing the α5 subunit. This interaction induces neuronal hyperpolarization and inhibits both synaptic transmission and LTP. Consequently, these actions form the fundamental mechanism responsible for its anesthetic properties and the accompanying short-term memory impairment [67,68,69,70,71]. The functional dynamics of GABAα receptors, along with their responsiveness to isoflurane, demonstrate notable dependence on both the concentration of isoflurane and the age of the subject. The dual dependency could be the underlying factor driving the starkly contrasting behavioral outcomes observed when isoflurane is administered under diverse conditions. Considering the concentration aspect, isoflurane elicits a biphasic modulation of GABAα receptors. At low concentrations, which correspond to sedative levels, isoflurane markedly facilitates the transmission of tonic inhibitory signals to hippocampal pyramidal neurons. It induces sedation by enhancing chloride ion currents [72,73], augmenting the binding affinity of GABA to its receptors, and prolonging channel open times [74]. A nuanced augmentation of this nature may be sufficient to modulate the excitatory/inhibitory (E/I) balance within neural networks. In the developing brain, this mechanism may transpire through the fortification of synaptic connections between critical neural nodes, such as the dorsomedial thalamic nucleus [75], resulting in enhanced fear memories in adulthood. Isoflurane primarily functions as an allosteric modulator at clinically pertinent anesthetic concentrations. Rather than directly activating ion channels, it amplifies the effect of GABA on its receptors. This amplification results in a significant prolongation of the decay phase of GABA-induced inhibitory postsynaptic currents (IPSCs), maintaining neurons in an extended state of intense inhibition, which ultimately results in the anesthetic outcome of loss of consciousness [47,76,77,78,79]. It is noteworthy that the mode of action shifts significantly when there is an unusual increase in the concentration of isoflurane. This agent further extends the decay time of IPSCs. Concurrently, its heightened channel-blocking capacity directly impedes receptor-mediated chloride currents, resulting in a substantial reduction in current amplitude [80]. Although the overall inhibitory effect appears to intensify, the risk of toxicity arises from receptor dysfunction. This offers insight into the paradoxical finding that young individuals may exhibit cognitive deficits, rather than improvements, following exposure to elevated concentrations.

Variations in GABAα receptor expression among different age demographics add complexity to the previously discussed concentration-dependent effects. In the developing brain, inhibitory synapses are robustly developed and stable, with neuroplasticity being notably high. Consequently, the potentiating influence of isoflurane on GABAergic inhibition may be both reversible and balanced. At lower concentrations, slight potentiation may potentially facilitate cognitive enhancement or the strengthening of fear memory by optimizing the excitatory/inhibitory balance [75]. Excessive inhibition or receptor blockage at elevated concentrations can exceed the boundaries of homeostatic regulation, leading to diminished cognitive function. Conversely, the hippocampus in an aging brain undergoes downregulation of the GABAα receptor’s alpha5 subunit. This downregulation weakens the GABAergic inhibitory signaling, thrusting neural networks into a state of disinhibition and heightened excitability [81,82]. At this juncture, the application of isoflurane can be undertaken, potentially exerting robust inhibitory effects despite the activity of other GABA receptors. This may precipitate acute oscillations between intense excitation and imposed inhibition, which are anticipated to disrupt the homeostasis within neural networks significantly. Additionally, given that the efficacy of inhibitory interneurons in the hippocampus and the mechanisms for GABA clearance in the prefrontal cortex are diminished in older adults, such enforced inhibition often triggers an overstimulation of the excitatory/inhibitory balance. Consequently, this may lead to sustained cognitive dysfunction [83,84,85]. It might also offer a fresh insight into the reason why older rodents have difficulty reaching the same level of cognitive improvement as younger rodents, even at low doses.

#### 4.1.2. Glycine Receptors

Glycine receptors (GlyR), which are ligand-gated chloride channels, primarily reside in the spinal cord and brainstem. When glycine binds to these receptors, it triggers the opening of the channel, resulting in an influx of chloride ions. This influx induces neuronal hyperpolarization, thereby facilitating the conveyance of inhibitory signals within the central nervous system. Isoflurane has been shown to allosterically modulate the function of glycine receptors. It does so by significantly enhancing the amplitude of chloride currents and prolonging the decay phase of these currents. Through these mechanisms, isoflurane augments postsynaptic inhibitory actions, a process believed to underlie its properties as a muscle relaxant, analgesic, and sedative [86,87].

The activity and level of glycine receptors exhibit pronounced age-related variability, akin to that observed in GABA receptors. In juvenile rats, the density of [3H] glycine-binding sites in regions such as the frontoparietal cortex, striatum, and thalamus is markedly elevated compared to the adult phase, where a notable decrease in these sites is observed, particularly in the striatum, hippocampal region CA3, and thalamus. As rats progress into middle and old age, a pervasive age-associated decline in the number of binding sites is evident across all studied brain regions [88]. The transition implies that glycine receptors exhibit significantly greater susceptibility to the aging process compared to NMDA receptors, which will be examined subsequently. It is crucial to note, however, that glycine receptors do not have a direct involvement in memory formation [89]. Still, their age-related level of expression as an inhibitory target of isoflurane can affect the neural reaction to anesthetics in older rodents, thus influencing cognitive recovery and neural network stability indirectly. It offers a fresh insight into neuro-pharmacology in explaining why older rodents are at high risk of developing post-anesthesia cognitive dysfunction [90,91].

### 4.2. Imbalance in the Excitatory System and Calcium Homeostasis

In addition to enhancing inhibitory transmission, isoflurane significantly influences excitatory signal transmission, a critical factor in moderating its cognitive effects. Primarily, isoflurane targets the NMDA receptor, with this regulatory mechanism demonstrating a pronounced age-dependence, thereby exacerbating cognitive decline associated with the aging brain [10].

#### NMDA Receptors

NMDA receptors are heterotetrameric ion channels composed of various combinations of GluN1, GluN2A-D, and GluN3A-B subunits. Isoflurane, acting as an antagonist, impedes calcium ion influx by directly binding to an internal ligand-binding site within the receptor. Notably, prolonged inhibition triggers compensatory mechanisms within the body, leading to increased receptor expression as a means to restore neural activity [92,93,94].

Isoflurane has a characteristic double effect due to age: young rodents have greater densities and functionality of NMDA receptors (especially the GluN2B subunit). In addition, young rodents have a completely developed blood–brain barrier and resting microglia, without a predilection towards neuroinflammation. In conjunction with the pronounced synaptic plasticity and homeostatic regulation inherent in the brain, the administration of isoflurane typically results in merely transient inhibition. The temporary augmentation of the NR2B subunit, observable post-anesthesia application, is generally perceived as an adaptive response [10] and might also be linked to a short-term cognitive enhancement. Eventually, the drug is quickly eliminated, neural circuits are restored, and downstream signaling goes back to normal. Thus, it does not usually leave a lasting effect [5,95,96].

The situation is markedly different in older rodents, however. The density and activity of NMDA receptors (especially the GluN2B subunit) in the cerebral cortex and hippocampus decline dramatically with age [97,98,99,100], causing an imbalance in the subunit expression. It causes decreased sensitivity to NMDA in the prefrontal cortex of the old brain [101]; the Short-Term Potentiation (STP) and the NMDA receptor dependent LTP are attenuated in the hippocampal CA1 region, whereas calcium channel-dependent LTP is enhanced in compensation [102,103,104,105,106]. Within this susceptible context, isoflurane induces an aberrant overexpression of NMDA receptors along with the NR2B subunit [106]. This maladaptive compensatory response fails to reinstate normal functionality and further disrupts calcium homeostasis, impedes the downstream p-ERK signaling cascade, and activates caspase cascades. In addition, the inflammatory aging that exists in elderly rodents gets intensified by isoflurane effects, which eventually cause a detrimental damage amplification effect, contributing to the mental deterioration.

Not only does this age-related variation explain the higher tendency of elderly rodents to suffer post-anesthesia cognitive impairment but also clearly defines the direction of specific interventions. Studies have shown that NR2B transgenic mice retain good learning and memory abilities even in a state of aging [107]. The mice in question naturally exhibit elevated levels of NR2B compared to typical mice. Consequently, upon exposure to isoflurane, this enhanced baseline expression may have directly mitigated the anesthetic’s inhibitory effects, thus averting the development of pathological “overcompensation.” This finding partially supports the previously discussed mechanism behind the excessive compensatory response noted in older adults.

In alignment with this observation, research conducted with age-matched mice has further illustrated that augmenting NR2B expression—either through the inhibition of Cdk5 or the upregulation of the transporter KIF17—can significantly bolster synaptic plasticity and enhance long-term potentiation, in addition to improving cognitive and memory functions [5,107,108]. Collectively, this body of evidence indicates that augmenting the expression of the NR2B subunit within the aging brain might constitute a pivotal approach for mitigating and addressing age-associated memory deterioration.

**Figure 4:**
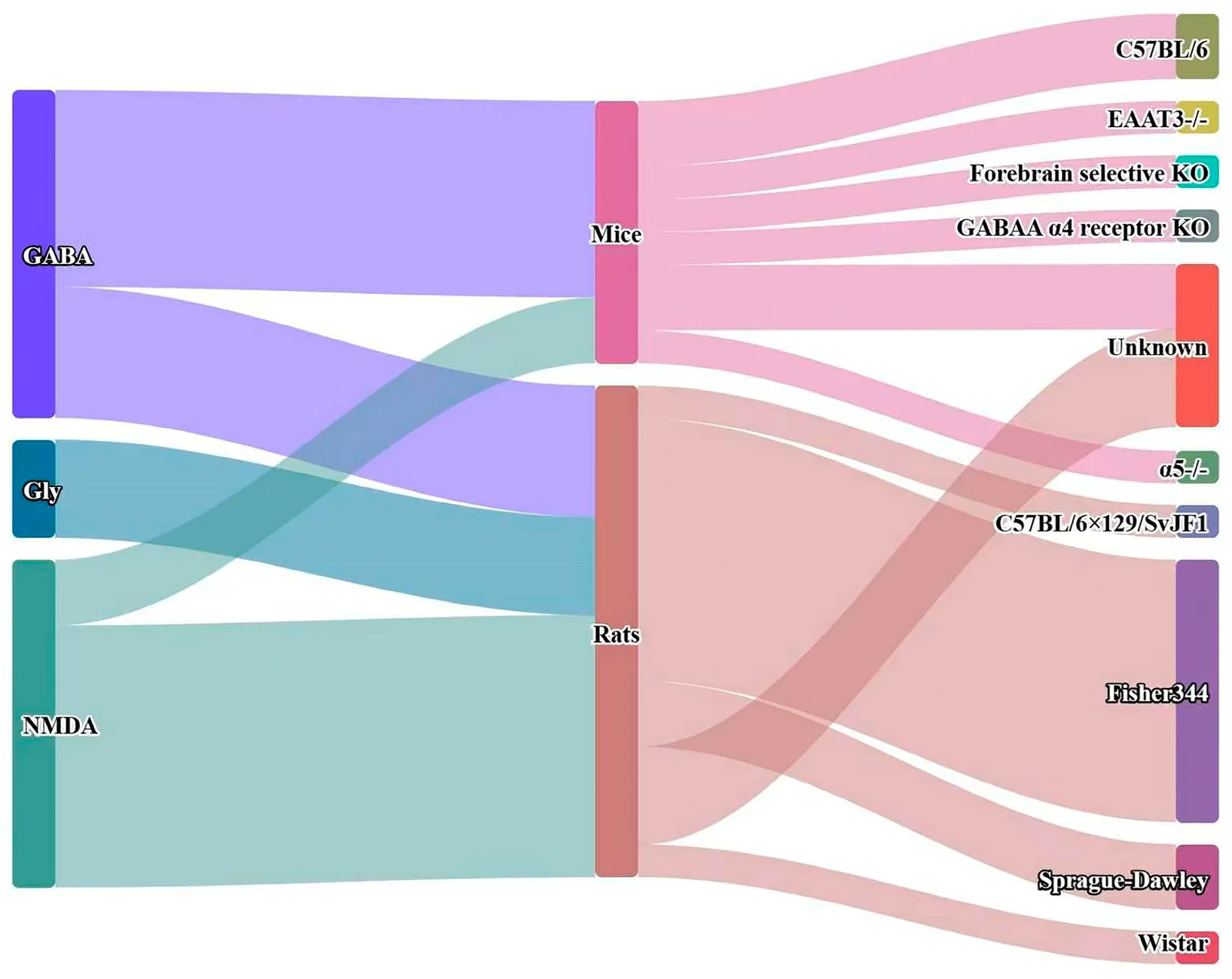
Sankey Diagram of Rodent Strain Distribution in GABA, Glycine and NMDA Receptor Studies. Sankey diagram showing the distribution of studies focusing on NMDA, glycine, and GABA receptors in rat and mouse models. The width of each stream represents the number of studies. An analysis of research trends reveals distinct differences between the two species. While most experiments in rats focus on NMDA receptors, studies in mice primarily investigate GABA receptors. This indicates that although these two rodent models share the same receptor pathways, they exhibit different preferences regarding their targets.

In addition to the classic receptor hypothesis, a growing body of evidence suggests that microtubules and cytoskeletal components may also be key pathways for volatile anesthetics [109,110]. Clinical findings indicate significant interactions between anesthetics and microtubule-stabilizing anticancer drugs, providing further support for this view [38,59,111,112,113,114]. Overall, the neuropharmacological effects of isoflurane depend on the regulation of multiple molecular targets. We hypothesize that the dual nature of these molecular targets likely leads to the diverse responses observed in experimental animals following isoflurane exposure. This finding may also offer new insights for future neurotherapeutic interventions. Based on the discussion of these multiple targets and to facilitate subsequent research, we have systematically reviewed and summarized the rodent models used in the relevant literature. See Figure 4 for details.

## 5. Intervention Strategies and Challenges in Translation

### 5.1. The Intersection of Therapeutic Agents/Molecular Targets with Core Pathways

**Figure 5:**
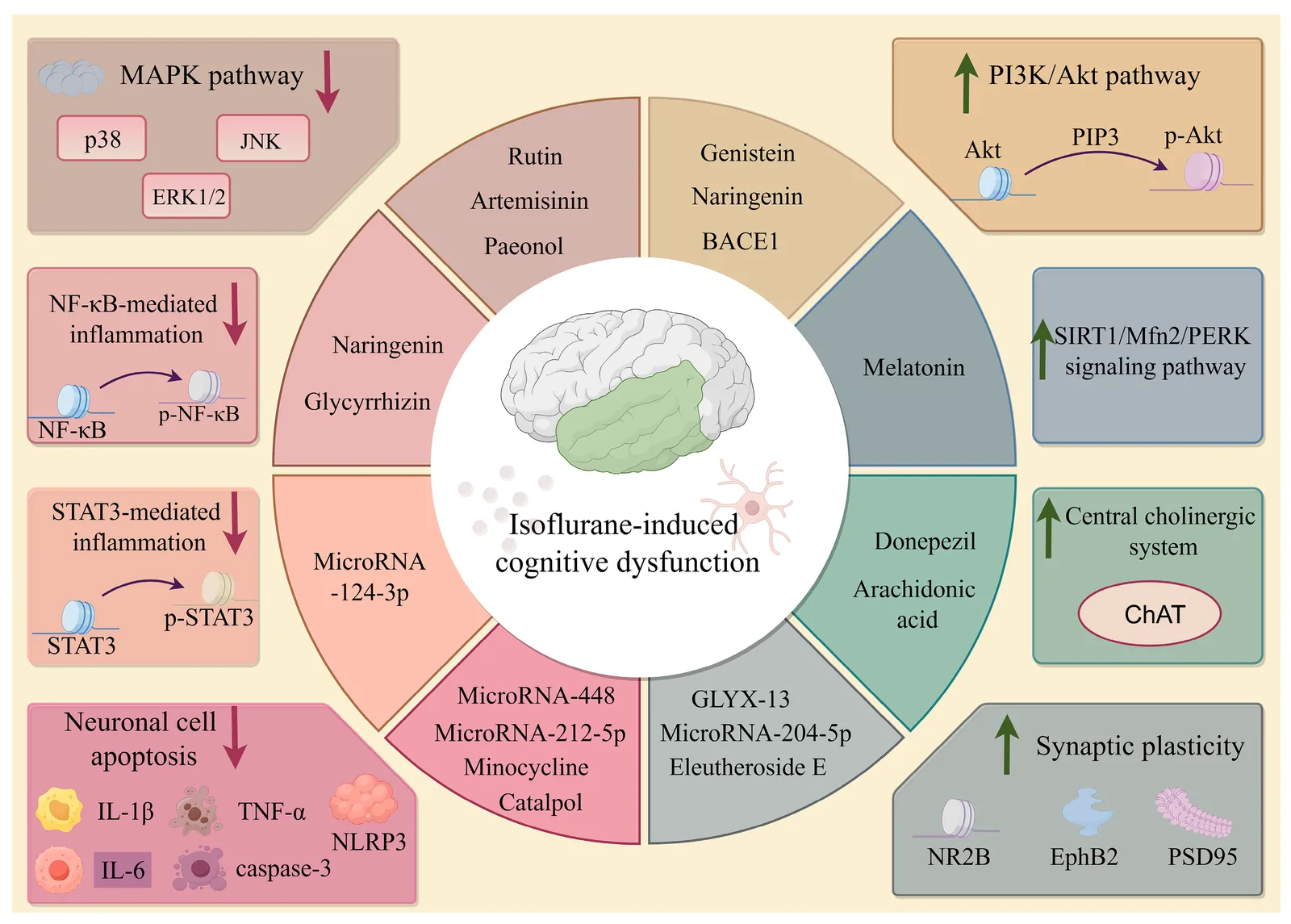
Network of multi-pathway intervention strategies for alleviating isoflurane-induced cognitive impairment. This figure illustrates the relationships between intervention strategies (inner circle) and key signaling pathways, molecular targets and their corresponding downstream biological effects (adjacent outer circle). Each drug is positioned adjacent to its primary pathway of action and color-coded accordingly; targets include the MAPK and PI3K/Akt signaling cascades, inflammatory regulators (NF-κB, STAT3, NLRP3), proteins associated with synaptic plasticity, apoptotic mechanisms, and miRNA-mediated regulatory axes. A green upward arrow indicates upregulation or activation, while a red downward arrow indicates downregulation or inhibition, and a horizontal arrow indicates phosphorylation. This network diagram provides a comprehensive overview of drug intervention strategies targeting isoflurane-induced cognitive impairment and neuroinflammation.

Based on the previously discussed multi-target network of isoflurane’s mechanism of action and the “dose–age-effect” model, researchers have developed intervention strategies targeting distinct stages of these pathways. These strategies fall into two main categories based on their targets: strategies that prevent neuronal apoptosis and strategies that protect synaptic plasticity. Table 4 and Table 5 provide an enumeration of the drugs and bioactive compounds with potential clinical significance for each category, as depicted in Figure 5.

Although various drugs are currently available to improve cognitive impairment induced by isoflurane, most interventions focus on only a few key pathological pathways. These pathways include the NF-κB/NLRP3 inflammatory axis, the MAPK signaling cascade, the PI3K/Akt survival pathway, mechanisms associated with oxidative stress, and factors regulating synaptic plasticity, such as PSD-95. Many compounds do not achieve their neuroprotective effects through entirely distinct mechanisms. Rather, they primarily achieve these effects by inhibiting neuroinflammation, reducing neuronal apoptosis, maintaining mitochondrial function, and restoring synaptic integrity.

In addition to the classic signaling pathways mentioned above, microtubules, a core component of the cytoskeleton, have been shown in recent years to be a direct target of isoflurane. Clinical evidence reveals significant interactions between microtubule-stabilizing chemotherapeutics and anesthetic responses in surgical patients [111]. Animal studies have further confirmed that isoflurane directly targets microtubules, leading to loss of consciousness in rats and mice [112,140], and that different microtubule-modulating drugs may exert opposing effects on sensitivity to isoflurane [113], further establishing the central role of microtubules in the mechanisms of anesthesia. Studies using computational modeling have also predicted that the potency of various volatile anesthetics, including isoflurane, is closely related to their microtubule-binding properties [114].

The clinical significance of the dose–age-effect framework extends beyond a purely mechanistic explanation; it may also provide a basis for personalized intervention strategies. Because cognitive outcomes result from the interaction between the dose of anesthesia and age, effective interventions may vary depending on the stage of development. In the developing brain, neuroprotective approaches may primarily target neuroinflammation, oxidative stress and programmed cell death pathways. However, in the aging brain, interventions may require additional strategies aimed at restoring synaptic plasticity and alleviating chronic neuroinflammatory responses. Consequently, the dose–age-effect framework can help select therapeutic targets more rationally based on the biological context of anesthetic exposure.

### 5.2. Priority Assessment and Challenges in Translational Medicine

**Figure 6:**
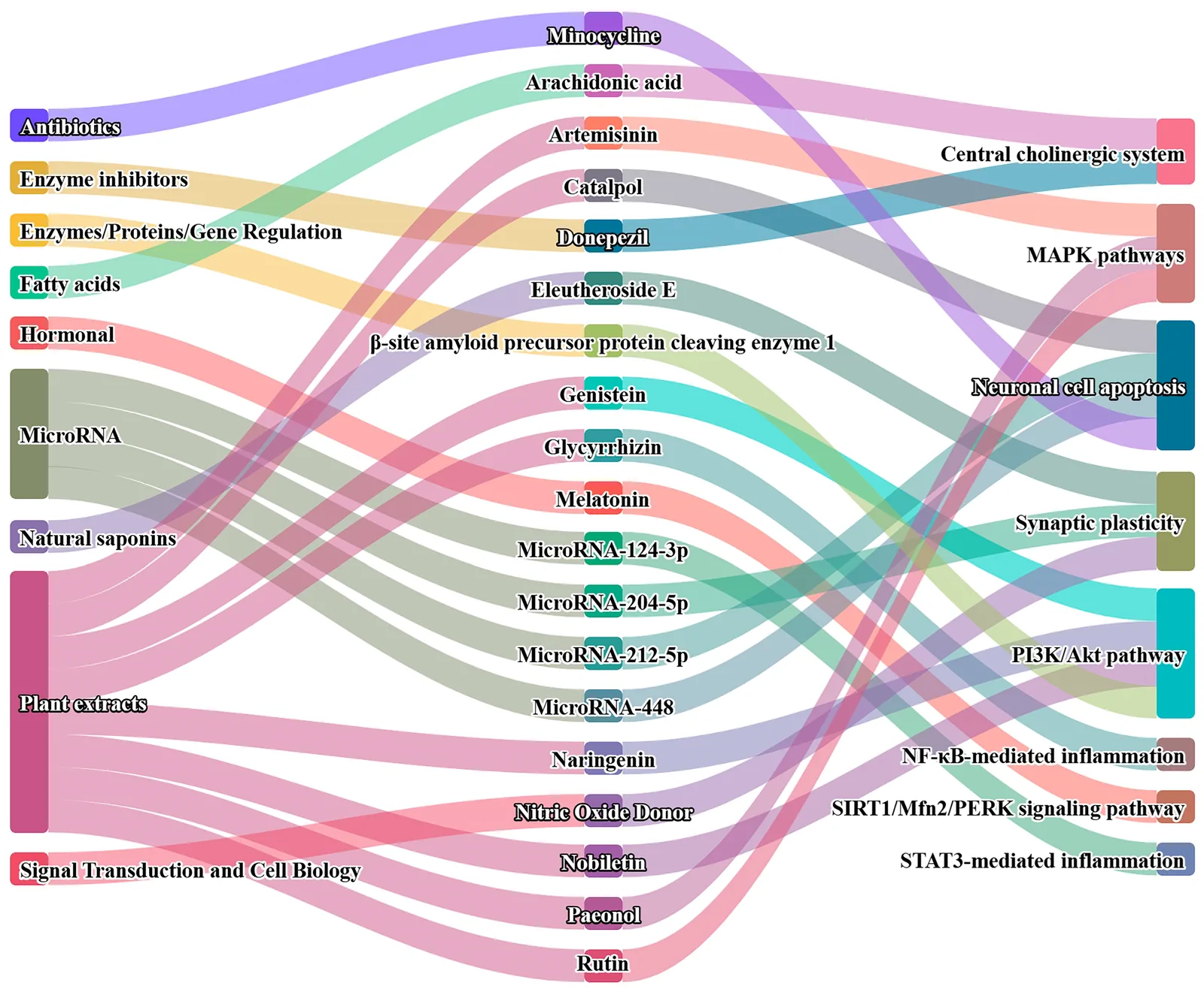
Correlation analysis of intervention strategies and multi-core regulatory pathways against isoflurane-induced cognitive impairment. This Sankey diagram illustrates the relationships between nine categories of intervention strategies and the eight core pathways involved in counteracting isoflurane neurotoxicity. The width of the lines represents the frequency of relevant studies. In terms of overall distribution, research on plant extracts covers a wide range of downstream signaling pathways, making it the intervention type with the broadest scope of current research; microRNAs and natural saponins are primarily focused on pathways that inhibit inflammation and neuronal apoptosis, with the existing literature largely centering on their regulation of inflammation and cell death; the MAPK and PI3K/Akt pathways, as well as synaptic plasticity, are currently among the most frequently studied core regulatory targets.

To provide a clear illustration of the distribution patterns of the molecular pathways associated with isoflurane-induced neurotoxicity across different intervention methods in existing studies, this study utilized a Sankey diagram to visualize and organize the data. (Figure 6).

From a clinical application perspective, significant differences exist among intervention strategies in various categories. Some active ingredients, such as donepezil, melatonin, and propofol, have already demonstrated clear clinical significance. However, the majority of natural products, microRNAs, and experimental signaling pathway modulators remain in the preclinical research stage. Therefore, intervention strategies targeting specific signaling pathways may be more clinically significant than individual potential drugs. In the development of future therapies, it is believed that targeting general key areas of mechanisms of action is more effective than focusing on individual active ingredients.

The results of the aforementioned preclinical studies appear promising. However, there are still many challenges involved in translating these findings from the laboratory to the clinical setting. Most of the treatments discussed so far have only been demonstrated to be effective in rodent models. There is a lack of validation studies in large animals and non-human primates. Furthermore, the response to isoflurane varies by animal species. Because the time interval between isoflurane administration and the start of treatment varies across studies, it is difficult to establish a standard clinical protocol. The safety profiles and blood–brain barrier permeability of most plant-derived active compounds are not well understood, and the risks of long-term application are unclear.

## 6. The Relationship Between Isoflurane and Cognitive Impairment-Related Diseases

### The Bidirectionality of Isoflurane’s Cognitive Effects and Its Clinical Association with Cognitive Impairment Disorders

Early exposure to anesthetic agents may pose a substantial risk of enduring neurodevelopmental toxicity, representing a notable public health challenge [141,142]. A comprehensive array of preclinical and clinical research underscores a significant correlation between surgical interventions coupled with anesthesia and the initiation and progression of cognitive decline. Clinically, anesthesia-related cognitive deficiencies typically manifest as perioperative neurocognitive disorder (PND), with postoperative delirium (POD) and postoperative cognitive dysfunction (POCD) being the predominant instances. In pediatric patients, such cognitive impairment may appear as neuropsychiatric developmental disorders, including autism spectrum disorder (ASD) or intellectual developmental delay, while in geriatric populations, it may elevate the risk of Alzheimer’s disease (AD) [143]. Specifically, Alzheimer’s disease is a chronic, progressive ailment characterized by memory deficits and other cognitive impairments [144]. It constitutes the most prevalent dementia form and is linked with amyloid plaque formation and neurofibrillary tangles [145,146,147]. This condition impacts 13% of the elderly demographic (individuals over 65 years) and comprises two-thirds of chronic neurodegenerative disease instances. Postoperative cognitive dysfunction (POCD) entails cognitive impairments, such as sustained memory deterioration and concentration difficulties, subsequent to anesthesia and surgical procedures [148]. Key risk factors encompass extensive and invasive surgeries, prolonged anesthesia durations, advanced age, and comorbidities like diabetes mellitus and hypertension.

Isoflurane, a widely utilized volatile anesthetic in clinical settings, exerts a dual influence on cognitive function, unveiling novel research avenues for conditions related to cognitive deficits. Notably, prolonged exposure to high doses of isoflurane has been identified as a substantial risk for developing PND, establishing a theoretical basis for refining anesthesia practices. Adjustments might involve reducing isoflurane’s dosage and exposure time in elderly and pediatric populations or combining it with neuroprotective compounds. Conversely, the cognitive-enhancing effects of low-dose isoflurane during developmental periods provide promising prospects for addressing neurodevelopmental disorders such as ASD and intellectual developmental delays. Early-stage clinical trials by the Guangzhou Women and Children’s Medical Center have corroborated the potential efficacy of this treatment approach. Thus, delving into the bidirectional cognitive effects of isoflurane and their foundational mechanisms could yield significant insights into the pathogenesis of neurocognitive disorders and guide the formulation of therapeutic interventions.

## 7. Conclusions

Conventional perspectives have predominantly highlighted the “neurotoxicity” associated with isoflurane. Nonetheless, this viewpoint inadequately explains the bidirectional effects demonstrated in various studies, notably the improvement in cognitive function after low-dose, short-duration exposure. To address this discrepancy, this review introduces a dose–age-effect framework. It posits that the cognitive outcomes observed in experimental subjects are influenced by the interplay between the isoflurane exposure parameters and the developmental stage of the rat brain. Under various conditions, isoflurane persistently engages a comprehensive array of pivotal signaling pathways, notably the NF-κB/NLRP3 inflammatory axis, the MAPK pathway, and the PI3K/Akt signaling cascade. These intricate pathways eventually coalesce onto fundamental downstream effector molecules, such as caspase-3, along with proteins associated with synaptic plasticity, including brain-derived neurotrophic factor (BDNF) and PSD-95. This observation implies that the dual effects of isoflurane originate from its capacity to modulate shared molecular pathways in a context-dependent manner. Furthermore, the mechanisms that drive specific pathological conditions provide further refinement to this conceptual framework. In the developmental brain, exposure to substantial doses particularly triggers several forms of regulated cell death, such as ferroptosis (via GPX4/SLC7A11), necrotic cell death (through RIPK1), and pyroptosis (mediated by NLRP3 inflammasome activation). This suggests a markedly heightened sensitivity to metabolic and inflammatory duress. Conversely, the aging brain displays a distinct vulnerability profile, characterized predominantly by an imbalance in the expression of the NR2B subunit and the α5 subunit of the GABAα receptor, inhibition of the CBS/H_2_S pathway, and increased vulnerability of the blood–brain barrier. Collectively, these elements contribute to a phenomenon of “damage amplification”.

Our hypothesis posits that the bidirectional cognitive effects of isoflurane are fundamentally governed by fluctuations in the equilibrium between the NR2B subunit of the N-methyl-D-aspartate receptor and the α5 subunit of the gamma-aminobutyric acid type A receptor. In the context of a developing brain, moderate inhibition might trigger compensatory upregulation of NR2B, consequently enhancing synaptic plasticity. Conversely, within an ageing brain, excessive or prolonged inhibition might result in maladaptive overexpression of NR2B and the initiation of apoptotic pathways. Consequently, the ratio of NR2B to GABAα5 could emerge as a potential biomarker for predicting individual vulnerability to cognitive outcomes induced by isoflurane.

The significance of this research lies in its synthesis of molecular and systems-level evidence within a cohesive framework. By employing a context-based model to elucidate the interaction between anesthetics and the brain, it transcends the limitations of a basic neurotoxicity model. This approach lays a theoretical foundation for optimizing anesthetic approaches specifically tailored to pediatric and geriatric populations. Furthermore, the introduced molecular axis presents potential avenues for novel therapeutic interventions in cognitive disorders, including ASD and AD.

Recent investigations into the cognitive implications of isoflurane exhibit certain constraints. Notably, this study exclusively considered literature in English, which we recognize could restrict the broader applicability of the results. Furthermore, it is important to emphasize that age and dose should not be interpreted as the sole determinants of isoflurane’s effects on cognitive function. The evidence reviewed here suggests that additional factors, such as gender, genetic background, and environmental stressors, may modulate the response to anesthesia and interact with age- and dose-dependent mechanisms. For example, it has been shown that isoflurane exposure during the neonatal period acts synergistically with chronic stress to alter fear-related behaviors, highlighting the importance of considering environmental context when interpreting cognitive outcomes. Furthermore, extensive clinical trials are imperative to deliver robust evidence for the successful clinical translation of findings. This research presents an innovative viewpoint on the judicious clinical use of isoflurane in addressing cognitive impairment-related disorders. Nonetheless, its prospective clinical significance requires confirmation through additional fundamental and clinical investigations.

## Figures and Tables

**Table 1:** Rat studies on isoflurane-induced cognitive effects.

Species	Age (in Weeks)	Related Parameters	Key Differences	Reference
Rat	1	4 h, 3.5% MAC	↓ Proliferation of progenitor cells, hippocampal dysfunction	[23]
Rat	8	4 h, 2.4% MAC	Proliferation of early-stage progenitor cells ↓; proliferation levels in the later stage ↑	[23]
Rat	25	2 h, 1.2 vol%	Cognitive function shows signs of improvement	[6]
Rat	78	2 h, 1.2 vol%	Cognitive function is significantly impaired	[6]
Rat	10	1 h, 1.5 vol%	LTP ↓, basal GABAergic (BLA) ↑; emotional disturbances↑ fear-related learning impairments ↑	[24]
Rat	87	2 h, 1.2 vol%	No significant effects on behavior, learning, motivation	[25]

Note: vol%, volume concentration; MAC, minimum alveolar concentration. ↓ = decreased; ↑ = increased.

**Table 2:** Mice studies on isoflurane-induced cognitive effects.

Species	Age (in Weeks)	Related Parameters	Key Differences	Reference
Mice	1	6 h, 1.4 vol%	ΔFosB (BLA and the DG of the hippocampus) ↑; consolidation of fear memories ↑	[27]
Mice	1	6 h, 0.75 vol%	cognitive function ↑; consolidation of fear memories ↑	[26]
Mice	8	4 h, 2.4 vol%	Caspase ↑; NR2B protein levels ↓; pERK1/2 to total ERK ↓; no significant cognitive changes	[9]
Mice	8	0.5 h, 0.7 vol%	NR2B (Acute and long-term) ↑ p-ERK1/2/total ERK ↑; Cognitive Abilities ↑	[9]
Mice	8	2 h, 0.7 vol%		
Mice	8	4 h, 1.4 vol%	caspase (Acute and long-term) ↑; NR2B ↓; pERK1/2 to total ERK (acute and long-term) ↓; cognitive impairment	[9]

Note: vol%, volume concentration; MAC, minimum alveolar concentration. ↓ = decreased; ↑ = increased.

**Table 3:** Common mechanisms underlying cognitive impairment caused by low- and high-dose isoflurane.

Institutional Level	Specific Mechanisms/Pathways	Key Figures/Events	Main Consequences
Basic inflammatory response	Inflammatory pathways	TLR2/NF-κB activation	Visible at both low and high doses; magnified at high doses
Fluctuations in stress signals	MAPK pathway	Changes in JNK/p38/ERK levels	Basic stress response, varying in severity
Changes in the epigenetic basis	HDAC/Acetylation Regulation	HDAC2/3 ↑, histone acetylation ↓	Apparent regulation present at both doses
Basic Synaptic Regulation	Enhanced tonic inhibition	Mediated by the GABAα receptor δ subunit	Common regulatory points of the excitation-inhibition balance
	Fluctuations in synaptic scaffold proteins	Altered PSD-95 expression	Common targets of synaptic structure interactions

Note: ↓ = decreased; ↑ = increased.

**Table 4:** Characteristics of Drugs/Molecules Used to Intervene in Isoflurane-Induced Neuronal Apoptosis.

Category	Drugs/Bioactive Molecules	Core Mechanisms of Action and Related Signaling Pathways/Targets	Reference
MicroRNA	MicroRNA-133b	Survival and anti-apoptotic capacity in hippocampal neurons ↑	[115]
	MicroRNA-448	Neuronal apoptosis ↓	[116]
	MicroRNA-212-5p	Neuronal apoptosis ↓	[117]
	MicroRNA-204-5p	Targets EphB2	[118]
	MicroRNA-124-3p	Targets STAT3	[119]
Fatty acids	Arachidonic acid (ARA)	Modulates cholinergic and Glu/GABA regulatory system	[120]
Anesthesia-related medications	Propofol	Neuronal apoptosis ↓	[121]
	Alfaxalone (ALF)	Neuronal apoptosis ↓	[122]
Plant extracts	Catalpol	Modulation of the NLRP3 inflammatory pathway	[4]
	Genistein	Regulates cAMP/CREB and BDNF-TrkB-PI3K/Akt signaling	[123]
	Rutin	Modulates the JNK and p38 MAPK pathways	[124]
	Nobiletin	Modulates Akt, Bax, p-CREB, and BDNF-related pathways	[125]
	Naringenin	Modulates the PI3K/Akt/PTEN signaling pathway; NF-κB-mediated inflammation	[126]
	Artemisinin	Regulates JNK/ERK1/2 signaling	[127]
	Paeonol	Modulates the JNK/ERK/p38 MAPK pathway	[44]
	Glycyrrhizin	Neuronal apoptosis ↓	[128]
Enzymes/Gene Regulation	β-site amyloid precursor protein cleaving enzyme 1 (BACE1)	PI3K/Akt pathway ↑	[129]
Antioxidants	Vitamin C	Neuronal apoptosis ↓	[130]
Antibiotics	Minocycline	IL-1β and caspase-3-mediated inflammatory	[131]
Hormonal	Melatonin	SIRT1/Mfn2/PERK signaling pathway	[132]

Note: ↓ = decreased; ↑ = increased.

**Table 5:** Characteristics of Drugs/Molecules That Intervene in Isoflurane-Induced Synaptic Plasticity.

Category	Drugs/Bioactive Molecules	Core Mechanisms of Action and Related Signaling Pathways/Targets	Reference
Receptor agonists/antagonists	Ramelteon	melatonin receptor-mediated pathways	[133]
	GTS-21	α7 nicotinic acetylcholine receptors ↑	[134]
	Ciproxifan	Blocks H3 histamine receptors	[135]
Enzyme inhibitors	Donepezil	ChAT ↑	[136]
Natural saponins	Eleutheroside E (EE)	Modulates the α7-nAChR-NMDAR pathway	[137]
Signal Transduction and Cell Biology	Nitric Oxide Donor	Mediates the N-methyl-D-aspartate receptor/PSD-95/neuronal nitric oxide synthase pathway	[138]
Peptide modulators	Glycine-site NMDA receptor modulator (GLYX-13)	NR2B/CaMKII/CREB signaling pathway	[139]

Note: ↑ = increased.

## Data Availability

No new data were created or analyzed in this study. Data sharing is not applicable.

## Abbreviations

The following abbreviations are used in this manuscript:

AD	Alzheimer’s Disease
Akt	Protein Kinase B
ALF	Alfaxalone
AMPK	AMP-Activated Protein Kinase
APP	Amyloid Precursor Protein
Aβ	Amyloid Beta
ARA	Arachidonic Acid
Bad/Bax	Bcl-2-Associated Agonist of Cell Death
BBB	Blood–Brain Barrier
BACE1	β-site Amyloid Precursor Protein Cleaving Enzyme 1
Bcl-2/Bcl-xL	B-Cell Lymphoma 2/B-Cell Lymphoma-extra Large
BDNF	Brain-Derived Neurotrophic Factor
BLA	Basolateral Amygdala
CA1/CA3	Cornu Ammonis Area 1/3 (hippocampus subregions)
CaMKII	Calcium/Calmodulin-Dependent Protein Kinase II
CBS	Cystathionine β-Synthase
CDC42	Cell Division Cycle 42
Cdk5	Cyclin-Dependent kinase 5
c-Fos	Cellular Immediate Early Gene Fos
CIRI	Cerebral Ischemia–Reperfusion Injury
CIS	Cerebral Ischemic Stroke
CREB	cAMP Response Element-Binding Protein
CVS	Chronic Variable Stress
DG	Dentate Gyrus
EE	Eleutheroside E
Egr-1	Early Growth Response Protein 1
ER	Endoplasmic Reticulum
ERK	Extracellular Signal-Regulated Kinase
ERK1/2	Extracellular Signal-Regulated Kinase 1/2
E/I balance	Excitation/Inhibition Balance
GABA	Gamma-Aminobutyric Acid
GABAα	Gamma-Aminobutyric Acid Type A Receptor
Gly	Glycine
GlyR	Glycine Receptor
GPX4	Glutathione Peroxidase 4
GTS-21	α7 Nicotinic Acetylcholine Receptor Agonist
HDAC	Histone Deacetylase
HDAC2/3	Histone Deacetylase 2/3
HSP90	Heat Shock Protein 90
H3R	Histamine H3 Receptor
IL-1β	Interleukin-1 Beta
IPAs	Inhibitors of Apoptosis Proteins
IPSCs	Inhibitory Postsynaptic Currents
JNK	c-Jun N-terminal Kinase
KIF17	Kinesin Family Member 17
LTP	Long-Term Potentiation
MAC	Minimum Alveolar Concentration
MAPK	Mitogen-Activated Protein Kinase
MDL	Mediodorsal Thalamus
miR	MicroRNA
mTOR	Mammalian Target of Rapamycin
MWM	Morris Water Maze
MyD88	Myeloid Differentiation Primary Response Protein 88
NF-κB	Nuclear Factor Kappa-light-chain-enhancer of Activated B Cells
NLRP3	NOD-like Receptor Pyrin Domain Containing 3
NMDA	N-methyl-D-aspartate
NR2A/NR2B	N-methyl-D-aspartate receptor subtype 2A/2B
nAChR	Nicotinic Acetylcholine Receptor
PAK3	p21-Activated Kinase 3
PERK	Protein Kinase R-like Endoplasmic Reticulum Kinase
PI3K	Phosphoinositide 3-Kinase
PND	Perioperative Neurocognitive Disorders
POCD	Postoperative Cognitive Dysfunction
POD	Postoperative Delirium
ASD	Autism Spectrum Disorder
PSD-95	Postsynaptic Density Protein 95
PTEN	Phosphatase and Tensin Homolog
RIPK1	Receptor-Interacting Protein Kinase 1
ROS	Reactive Oxygen Species
SD rats	Sprague–Dawley Rats
SIRT1	Sirtuin 1
SLC7A11	Solute Carrier Family 7 Member 11
STAT3	Signal Transducer and Activator of Transcription 3
STP	Short-Term Potentiation
TLR2	Toll-Like Receptor 2
TrkB	Tropomyosin Receptor Kinase B
TUNL	Trial-Unique Nonmatching-to-Location
ULK1	Unc-51 Like Autophagy Activating Kinase 1
VEGFR2	Vascular Endothelial Growth Factor Receptor 2
Xc^−^ system	Cystine/Glutamate Antiporter System
ΔFosB	Delta FosB Transcription Factor
